# Supplementary material for: Dysbiosis of the Fecal and Biliary Microbiota in Biliary Tract Cancer
Source: Cancers (Basel). 2022 Oct 31;14(21):5379. doi: 10.3390/cancers14215379 (PMC9653963; doi:10.3390/cancers14215379)
Supplement: Supplementary file 1 [file cancers-14-05379-s001.zip › cancers-1912701-supplementary.pdf]

Figure S1

(A)*pks* genomic island and corresponding primers region

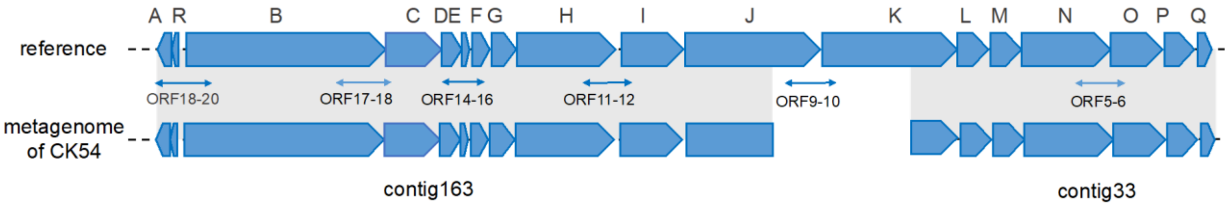

(B) BLAST identity against the query (%)

clbA	*clbR*	*clbB*	*clbC*	*clbD*	*clbE*	*clbF*	*clbG*	*clbH*	*clbI*	*clbJ**	*clbK**	*clbL*	*clbM*	*clbN*	*clbO*	*clbP*	*clbQ*
100	100	99.97	100	100	100	100	100	99.94	100	100	100	100	100	99.93	99.88	100	100

Figure S1. Colibactin related genes (*pks* genomic island) found in the metagenome data of cultured bacteria isolated from the bile juice. (A). *pks* genomic island and corresponding primers region. Alphabet above the reference genes indicate *clb*A to *clb*Q. Two direction arrow indicates primer region to detect the *pks* island as previously reported by Nougayrède, J.P. et al. [21]. (B). BLAST identity against the query (%) *Percentage in which located in the contigs of CK54 metagenome.

Figure S2

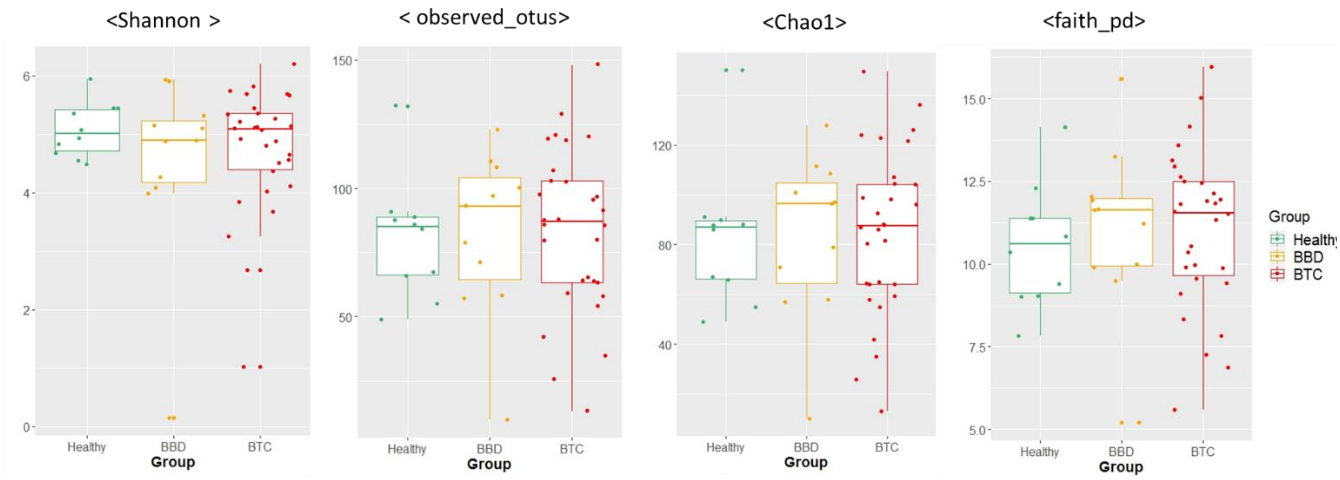

Figure S2. The alpha diversity based on the Shannon, observed_otus, Chao1 and faith_pd indices in each group.

Figure S3

＜Weighted UniFrac distance＞ ＜Unweighted UniFrac distance＞

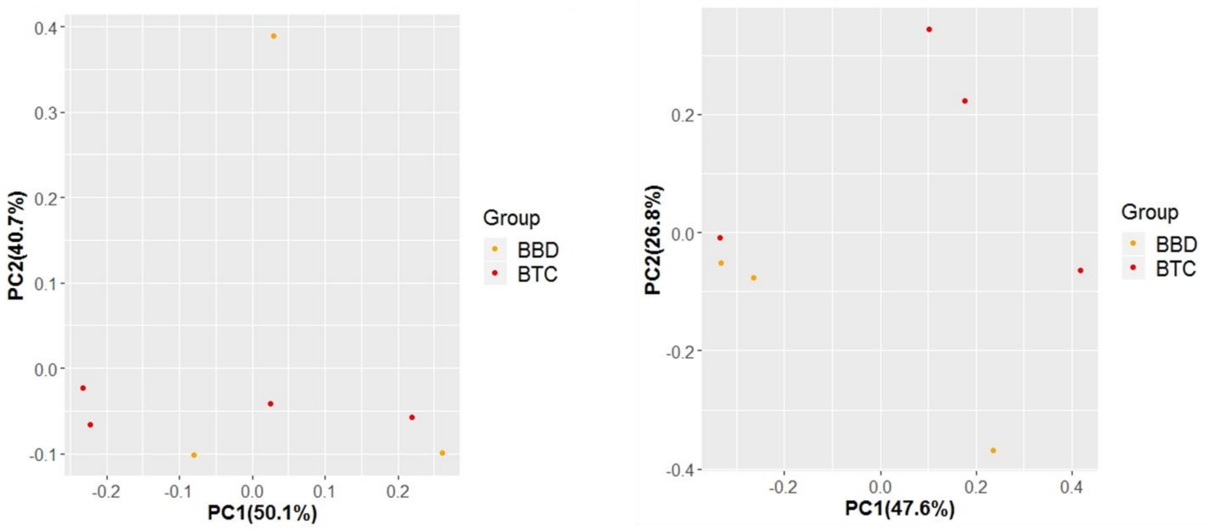

p=0.629 p=0.771

Figure S3. Weighted and unweighted UniFrac PCoA of the bile microbiota in patients with BBD and BTC after the amplification of 16S rRNA genes in four of eight patients with BTC and in three of ten patients with BBD.

Figure S4

(A) (B) (C)

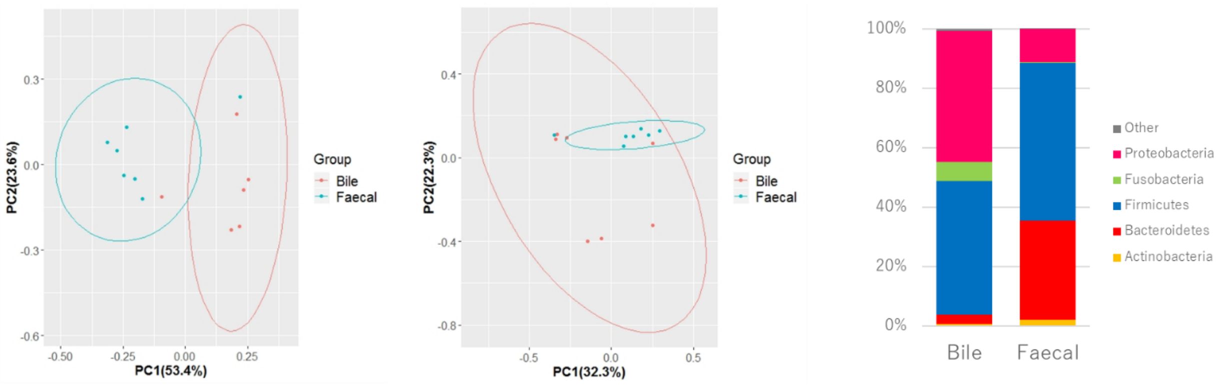

＜Weighted UniFrac distance＞ ＜Unweighted UniFrac distance＞

Figure S4. (A) Weighted and (B) unweighted UniFrac PCoA of the bile and fecal microbiotas in patients with BBD and BTC. (C) Composition of the bile and fecal microbiota at the phylum level.
